# Interleukin-8 drives epithelial-mesenchymal transition of human carcinomas

**DOI:** 10.1186/2051-1426-1-S1-P187

**Published:** 2013-11-07

**Authors:** Romaine I Fernando, Duane H Hamilton, Bruce Huang, Claudia Palena

**Affiliations:** 1 Laboratory of Tumor Immunology and Biology, Center for Cancer Research, NCI, NIH, Bethesda, MD, USA

## 

The phenomenon of epithelial-mesenchymal transition (EMT) has been proposed as a relevant event during carcinoma progression. Tumor EMT drives the phenotypic conversion of polarized, epithelial tumor cells into highly motile, mesenchymal-like cells, and promotes tumor dissemination and metastasis and the acquisition of tumor resistance to various therapies. In order to investigate the potential role of EMT in the modulation of the tumor microenvironment as well as to identify soluble factors that could initiate and/or maintain tumor EMT, isogenic tumor cell pairs with epithelial vs. mesenchymal-like features were generated via modulation of the expression of an EMT driver, the T-box transcription factor Brachyury. A characterization of the pattern of soluble factors secreted by each cell type showed that acquisition of a mesenchymal-like phenotype by epithelial tumor cells was associated with secretion of multiple cytokines and chemokines and, in particular, markedly enhanced the expression of the interleukin-8 (IL-8)/IL-8R axis. Interference with IL-8 signaling via IL-8 neutralization or blockade of IL-8 receptors was able to revert the invasive, mesenchymal phenotype of Brachyury-high tumor cells towards a non-invasive, epithelial one, therefore indicating that upregulation of the IL-8/IL-8R axis is critical for the maintenance of tumor EMT. The ability of IL-8 to induce EMT was also demonstrated on a panel of epithelial cancer cell lines that showed increased expression of mesenchmal markers, cell migration and invasion in response to recombinant IL-8. The correlation between Brachyury, tumor EMT and IL-8 expression was also investigated with various lung and colon cancer cell lines with acquired resistance to EGFR inhibition. These resistant cells exhibited changes characteristic of EMT, had high levels of brachyury and other EMT markers expression, and secreted significantly higher level of IL-8 compared to parental cells. Collectively, our results emphasize the role of the IL-8/IL-8R axis in the modulation of human tumor EMT and form the rationale for further investigations on the modulation of IL-8 signaling in the tumor microenvironment as an approach against carcinoma progression.

